# Communication and wiring in the cortical connectome

**DOI:** 10.3389/fnana.2012.00042

**Published:** 2012-10-16

**Authors:** Julian M. L. Budd, Zoltán F. Kisvárday

**Affiliations:** ^1^Department of Informatics, University of SussexFalmer, East Sussex, UK; ^2^Laboratory for Cortical Systems Neuroscience, Department of Anatomy, Histology and Embryology, University of DebrecenDebrecen, Hungary

**Keywords:** axon, cerebral cortex, communication, connectome, dendrite, networks, optimization, Ramón y Cajal

## Abstract

In cerebral cortex, the huge mass of axonal wiring that carries information between near and distant neurons is thought to provide the neural substrate for cognitive and perceptual function. The goal of mapping the connectivity of cortical axons at different spatial scales, the cortical connectome, is to trace the paths of information flow in cerebral cortex. To appreciate the relationship between the connectome and cortical function, we need to discover the nature and purpose of the wiring principles underlying cortical connectivity. A popular explanation has been that axonal length is strictly minimized both within and between cortical regions. In contrast, we have hypothesized the existence of a multi-scale principle of cortical wiring where to optimize communication there is a trade-off between spatial (construction) and temporal (routing) costs. Here, using recent evidence concerning cortical spatial networks we critically evaluate this hypothesis at neuron, local circuit, and pathway scales. We report three main conclusions. First, the axonal and dendritic arbor morphology of single neocortical neurons may be governed by a similar wiring principle, one that balances the conservation of cellular material and conduction delay. Second, the same principle may be observed for fiber tracts connecting cortical regions. Third, the absence of sufficient local circuit data currently prohibits any meaningful assessment of the hypothesis at this scale of cortical organization. To avoid neglecting neuron and microcircuit levels of cortical organization, the connectome framework should incorporate more morphological description. In addition, structural analyses of temporal cost for cortical circuits should take account of both axonal conduction and neuronal integration delays, which appear mostly of the same order of magnitude. We conclude the hypothesized trade-off between spatial and temporal costs may potentially offer a powerful explanation for cortical wiring patterns.

## Introduction

“That apparent disorder of the cerebral jungle, so different from the regularity and symmetry of the spinal cord and of the cerebellum, conceals a profound organization of the utmost subtility which is at present inaccessible”

(p. 395, Cajal, 1937).

Communication has been defined as the flow of information between a transmitter, generating a signal, and a receiver, reconstructing a signal after its passage through a noisy channel (Shannon, 1948). In his theory of dynamic polarity, Cajal (1899) had correspondingly divided a neuron, the fundamental unit of the brain, into three functional compartments: a receptor apparatus (soma and dendrites), an emission apparatus (axon), and a distribution apparatus (terminal axon arbor). Significantly, Cajal's inferences about how axonal and dendritic wiring are used to communicate derived from anatomical data only. Physiological experiments confirmed Cajal's inferences concerning neural communication: an action potential (signal) generated by one neuron propagates along its axon and via a noisy synaptic connection (channel) induces a response in the soma and dendrites of other neurons (see Purves et al., 2007). The notion of individual neuron polarity, though modified, remains a foundation of our understanding of neural communication in cerebral cortex (see DeFelipe, 2010). Mapping cortical connectivity is, therefore, vital to defining the channels of information flow underlying cortical function in both health and disease.

Recent technical advances now offer significant improvements in mapping the apparent disorder of the “cerebral jungle” across a range of spatial scales. Large-scale serial electron microscopy (EM) of gray matter volumes (<1 mm^3^) can be used to map the fine structure of cerebral cortex (Mishchenko et al., 2010; Bock et al., 2011). Trans-synaptic viral tracing methods now make it possible to visualize multiple stages of synaptic connectivity (Wickersham et al., 2007). Combinatorial fluorescent protein labeling methods are used to separately color the processes of many individual neurons simultaneously to aid multiple axon tracing (Lichtman et al., 2008). Combined magnetic resonance imaging (MRI) techniques are now used to reconstruct the whole cortico-cortical pathway network for an individual brain *in vivo* (Hagmann et al., 2010). Thus, the future promises to yield far more mapping data concerning cerebral cortex.

Yet mapping cortical connectivity will not in itself tell us how cerebral cortex works (see Douglas and Martin, 2011). Indeed, even with these technical advances, the huge number of neurons and synapses per cortical hemisphere make constructing a whole map of synaptic connections or *connectome* impractical (Lichtman et al., 2008; DeFelipe, 2010). From available mapping data we need to discover the general organizing principles of the “cerebral jungle” and to infer what purpose these principles may serve in terms of cortical function.

To explain brain design, Cajal (1899) proposed that neuronal morphology was regulated by distinct organizing principles that sought to separately conserve cellular material (“wire”), conduction delay, and brain volume. Contemporary research on brain design has focussed predominantly on the wiring minimization principle (Mitchison, 1991; Cherniak, 1994; Chklovskii et al., 2002). Strong claims have been made that this organizing principle alone can explain, for example, the intracortical wiring underlying functional maps (Koulakov and Chklovskii, 2001). Recent studies suggest, however, that individual cortical neuron morphology (Budd et al., 2010; Cuntz et al., 2010) and neural networks are not organized by wire minimization only (Ahn et al., 2006; Kaiser and Hilgetag, 2006; Bassett et al., 2010).

We have previously proposed a multi-scale wiring principle for optimizing neuronal network communication in cerebral cortex (Budd et al., 2010). This hypothesis states that the conservation of cellular material (construction cost) is traded-off against the need to minimize conduction delay (routing cost). Indeed, for a modest excess of cellular material, this trade-off promotes precise and rapid communication in cerebral cortex, which has implications for our understanding of neural coding and synchrony (see Uhlhaas et al., 2009).

The main purpose of this article is to critically review recent evidence to discover how well this hypothetical wiring principle may explain cortical connectivity across different spatial scales. The article is not intended as a review of the cortical connectome approach *per se* but it does examine the utility of this framework to help evaluate this and other hypothetical wiring principles. We begin with a brief introduction to spatial networks and its application to different levels of cortical organization before evaluating evidence relating to the hypothesis.

## Spatial cortical networks: neurons, circuits, and pathways

Graph theory is a powerful technique for the mathematical abstraction of real world problems (see Newman, 2010). Box 1 offers a short introduction to relevant graph theory concepts and notation. Briefly, in a *network* each distinct entity of a given system is represented by a single *vertex* and the pairwise relations and processes between these entities is represented by an *edge* (see Box 1; for further details, see Cormen et al., 2001). The network configuration describes all possible paths of information flow within the system. A graph theoretic approach is applicable, therefore, if a system can be viewed as a collection of distinct yet inter-related objects.

Box 1 **Graph Theory.**

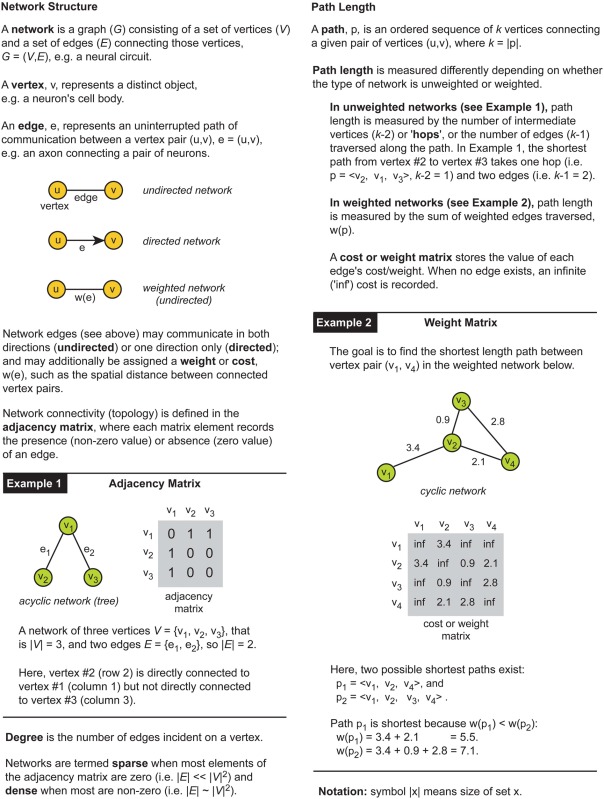

Neural systems can be decomposed into distinct objects and pairwise relations. For example, pre- and post-synaptic neurons are related yet distinct objects in a neural system as are, at a more basic level, the individual branches of a neuron's axonal or dendritic tree. But applying graph theory to model a neural system naturally requires assumptions to be made about the system's architecture. Characterizing an entire axonal pathway by a single edge, for instance, does not capture how information is distributed by the divergence/convergence of presynaptic axons in the target structure. Hence, it is important to be mindful of model assumptions and granularity when making inferences about cortical function from network models.

To construct a realistic biological network, the entities and pairwise relations in the biological system must be mathematically defined using available empirical evidence. When existing knowledge is insufficient or conflicting, however, it is necessary to either exclude certain system properties from consideration or make explicit assumptions regarding the system to resolve the issue. Once constructed, measures can be taken to describe the characteristics of the biological network and the results compared with those of artificial networks generated using hypothesized principles of organization. The degree of similarity between artificial and biological network characteristics can then be used to determine whether the hypothesized organizing principle merits further investigation, requires modification, or should be rejected. Here, we focus on spatial cortical networks, where vertices and edges have a physical correspondence to the anatomy of cerebral cortex. While not ignoring the importance of other relevant parameters, for reasons of available data we concentrate on two main costs in neuronal communication: conduction delay and cellular material.

### Spatial networks

A *spatial network* is a graph whose vertices have spatial coordinates and where measurements are taken with respect to their physical space (Barthélemy, 2011). In spatial cortical networks, each vertex represents a distinct neural feature in cerebral cortex with anatomical coordinates and each edge represents an uninterrupted path of communication between a vertex pair.

In an unweighted spatial network, analysis is directed toward understanding the relationship between space and topology in a given system, e.g., what connectivity patterns exist between particular groups of neurons? In the last decade or so, networks whose topology is neither entirely regular nor entirely random—*complex networks*—have generated considerable interest because of their ability to account for the organization of large-scale biological, physical, and social system using simple connectivity rules (Newman, 2010). In s*mall world* complex networks, for instance, the average path length between any given vertex pairs is reduced by incorporating a small percentage of long-distance connections in a network of mostly short-range regular connections (Watts and Strogatz, 1998). In *scale-free* complex networks, the vertex degree (see Box 1) distribution is described by a power law in which there are a small fraction of highly connected vertices called *hubs* (Barabási and Albert, 1999). Analyzing network partitions, *subgraphs*, may reveal further structural complexity. The conditional probability of a vertex pair being additionally connected to a third vertex is termed *clustering*, which is high in complex but low in random networks (Watts and Strogatz, 1998). Relatedly, when a given set of vertices has more connections in common than with other vertices, the subgraph is considered a (vertex) *community* or *module* (Girvan and Newman, 2002). Subgraphs with frequently repeating patterns of connectivity are termed *network motifs* (Milo et al., 2002).

In a weighted spatial network, analysis is focused on the relationships among cost, space, and topology, e.g., how much does a specific spatial and/or topological arrangement of neural features cost? Global network metrics are often used to address such questions. The total cost of the network is equal to the sum of all weighted network edges. For example, when edge cost is proportional to the Euclidean distance between a vertex pair the total cost gives the spatial *construction cost* of the network. In this article, distance will refer to Euclidean distance unless otherwise stated. A complementary metric is global *routing cost*, which is the average or total path length of a network. There are two alternative measures of routing cost. Latora and Marchiori (2001) have defined network *efficiency* as the inverse of the shortest path length and the average efficiency over all vertex pairs as a measure of global efficiency. Gastner and Newman (2006) have proposed *route factor* as a measure of efficiency for trees, the mean of metric path length divided by radius from root to all vertices.

**Figure 1:**
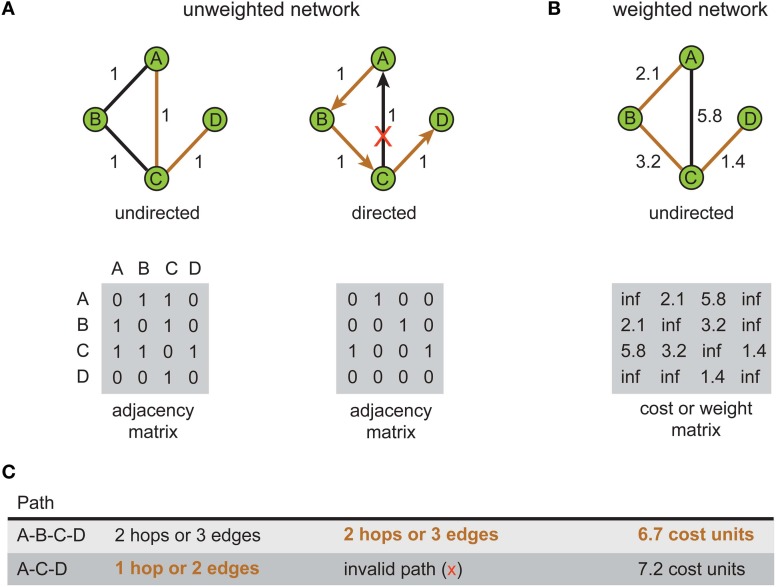
**Shortest path for the same problem can be different depending on the type of network representation used.** An example network consists of four labeled vertices A, B, C, and D. The aim is to find shortest path between vertex A to vertex D. **(A)** Unweighted network representation. The topology of undirected (left) and directed versions (right) is shown graphically (top) with their corresponding adjacency (connectivity) matrices below (bottom). Brown lines show shortest paths. Red cross indicates a counter-directional edge, which creates an invalid path from vertex A to vertex D. **(B)** Weighted network representation. Graphical representation (top) of an undirected weighted graph with values of weights (distance) shown next to edges and recorded in the cost or weight matrix below (bottom). Note in the cost or weight matrix the absence of an edge is recorded as an infinite cost (“inf”) while in adjacency matrix it is recorded as zero. **(C)** Summary table for path length results corresponding to each type of network. Shortest paths are shown in bold brown text.

How a system is represented graphically influences what can be inferred about its communication or processing characteristics. Figure 1 illustrates this point by comparing the shortest path length in unweighted (Figure 1A) and weighted network representations (Figure 1B) for the same toy problem. Recall that path length is measured differently between weighted and unweighted networks (see Box 1). In the unweighted representation, the use of directional edges removes one of two possible paths connecting vertex A to vertex D (Figure 1C). So if the directions of information flow were known for the system but not incorporated in the network model, inferences concerning the directness of communication would be distorted; the undirected network would allow information to flow along paths that were impossible in the real system. Similarly, the shortest length path in the unweighted network representation (Figure 1A, left) is the one with the fewest hops whereas in the weighted network representation of the same system (Figure 1B) it is the path with the lowest total cost (see Figure 1C). This means that the choice of graphical representation can for the same system identify different shortest paths, though this choice may be dictated by available data.

**Figure 2:**
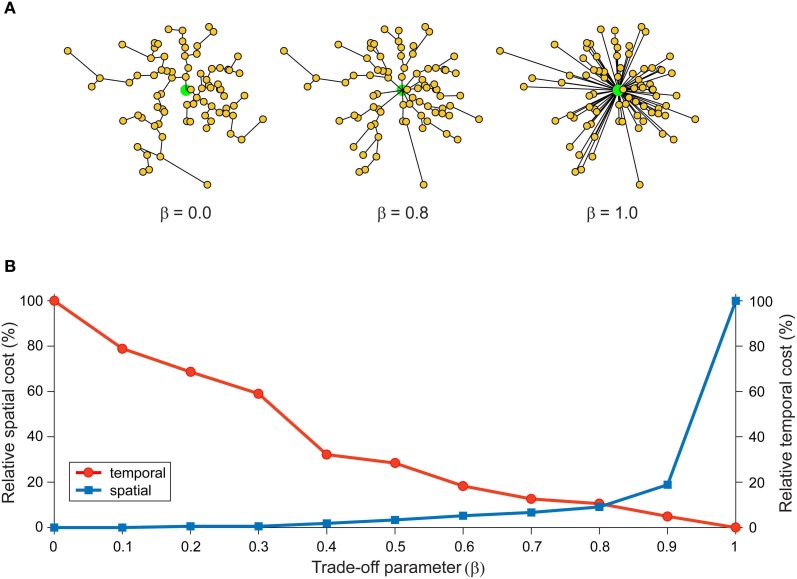
**Spatial and temporal cost trade-off alters arbor morphology.** An example network consists of 80 labeled vertices (small yellow filled circles) plus a root vertex (large green filled circle). Here, total wiring cost = spatial cost + (β × temporal cost), where the parameter β, which varies between 0 and 1, is used to trade-off spatial construction cost against temporal routing cost. **(A)** Artificial arbor structures optimized for different values of a cost trade-off parameter, β = 0.0 (spatial cost optimization, left), 0.8 (mixed cost optimization, middle), and 1.0 (temporal cost optimization, right). **(B)** Relative communication costs vary as a function of the trade-off parameter. Relative spatial cost (wire length) increases with β rapidly when β > 0.8, while relative temporal cost (path length) steadily decreases with β. Costs at equilibrium around β = 0.8. Artificial arbors were generated using Gastner and Newman (2006) algorithm.

In network design, simultaneously minimizing both construction and routing costs is considered an intractable (NP-hard) optimization problem because these are conflicting objective functions (Hu, 1974; Alpert et al., 1995; Khuller et al., 1995; Wu et al., 2002; Gastner and Newman, 2006). Figure 2 illustrates how a trade-off between these conflicting objective functions affects the structure of a spatial network. Here, optimizing total weight (construction cost) only leads to a *minimum spanning tree* (Figure 2A, left) or, if additional vertices are inserted, a *Steiner minimal tree* design (Garey and Johnson, 1979). In contrast, optimizing average/total path length only (routing cost) generates a *star tree* (Figure 2A, right), where there is direct connection from a central hub to each remaining vertex to create a hub-and-spoke design. Instead, a suboptimal minimization of construction cost permits a low routing cost (Figure 2A, middle). Figure 2B shows the relative change in communication costs in this spatial network for different values of β, a parameter that trade-offs spatial construction cost against temporal routing cost. When β = 0 then spatial cost is minimized and temporal cost maximized. Whereas when β = 1, the situation is reversed. Between these extremes, temporal cost decreases monotonically with increasing β while simultaneously spatial cost increases slowly until after the equilibrium point (β = 0.79) when it increases rapidly.

Here, the purpose of network analysis is to generate experimentally testable hypotheses to help advance our understanding of cortical organization and dynamics in health and disease (Bassett and Bullmore, 2009; Sporns, 2011; Leergaard et al., 2012—see *Frontiers in Neuroinformatics* Research Topic “Mapping the connectome: Multi-level analysis of brain connectivity”). To date, all network analysis suggests cortical connectivity has non-random, complex network characteristics (Sporns, 2011). In the following parts, we will discuss results obtained with multi-scale spatial analysis of cortical organization.

### Spatial scales of cortical organization

A *connectome* is a graph theoretic construct used to describe neural architecture at different spatial scales in terms of neural elements (vertices) and neural connections (edges) (see Sporns et al., 2005). Ideally, each edge should be annotated with a range of associated properties to completely describe its anatomical and physiological connection characteristics including axonal length and conduction delay. In the Human Connectome proposal, Sporns et al. (2005) argued that the organization of cerebral cortex could be viewed at three distinct spatial scales: *microscopic* (micron spatial resolution of the processes of individual neurons and synapses), *mesoscopic* (hundreds of micrometers spatial resolution of cortical columns and local circuits), and *macroscopic* (millimeter spatial resolution of brain regions and pathways). This framework elegantly utilizes the generality of graph theory to abstract anatomical entities and their relationships at different spatial scales of cortical organization. To understand brain structure as a whole, DeFelipe (2010) argues we need to possess connectomes for each spatial scale.

But the term “connectome” may be used too loosely (Kasthuri and Lichtman, 2007; DeFelipe, 2010). EM is required to confirm the presence of a synaptic connection (Peters et al., 1991) otherwise putative synaptic connectivity can only be inferred from the close spatial proximity such as axonal-dendritic membrane apposition (microscopic scale) or regional termination pattern (macroscopic scale) as is done in most studies using confocal microscopy. Consequently, a connectivity (adjacency) matrix constructed from axonal tracing but lacking ultrastructural confirmation might more accurately be referred to as a “projectome” (Kasthuri and Lichtman, 2007). Additionally, a connectome whose connectivity has been confirmed by EM might better be called a “synaptome” (DeFelipe, 2010). For the sake of simplicity, we use the term “connectome” here to mean the accurate structural description of connected neural elements.

The Human Connectome scheme has some degree of correspondence with the Levels of Brain Organization approach (Shepherd, 1990). In this the top level is concerned with mapping *systems and pathways level*, which relates directly with the macroscopic scale. The next level describes the *local circuit level*, defined as regional groups of interconnected neurons, which matches with the microscopic scale. However, the correspondence breaks down because the levels approach appears to lack a mesoscopic scale and the Connectome scheme lacks both a *neuron level*—discrete nerve cells as morphological entities with integrative properties—and a *microcircuit level*—stereotyped patterns of synaptic connections forming neuronal subunits. While the advantages of having a well-defined mesoscopic scale in cerebral cortex are clear (Bohland et al., 2009), no precise, and universal defintion currently exists (Horton and Adams, 2005; da Costa and Martin, 2010; Rockland, 2010). Here the problem lies in the fact that there is no consensus what should be the measure of dimension of mesoscopic connections.

## Communication costs at different spatial scales

**Figure 3:**
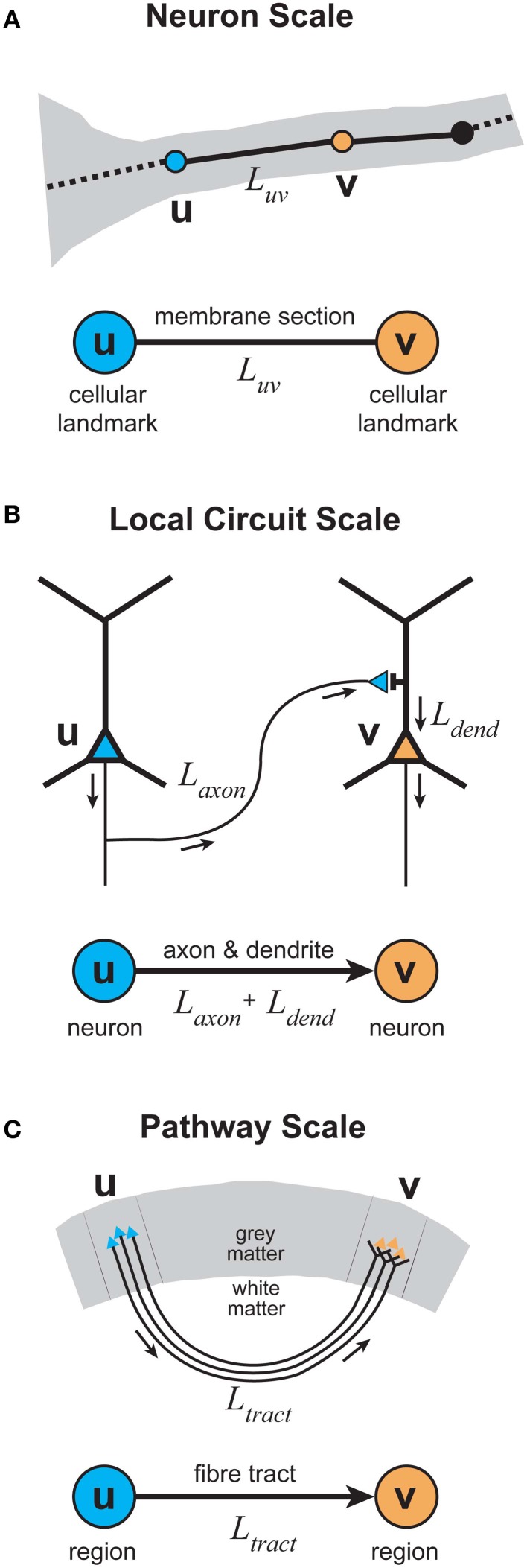
**Elementary graphical representations of cortical organization at different spatial scales. (A)** *Neuron scale*. Each vertex represents the location of a cellular landmark obtained from the 3D reconstruction of individual axonal or dendritic arbors (e.g., location of the presynaptic terminal boutons) with an undirected edge representing the section of membrane linking these vertices either by the actual path length or the direct distance between a vertex pair. **(B)** *Local Circuit scale*. Each vertex represents the somatic location of a single neuron with a directed (or undirected) edge representing the sum of the axonal and dendritic lengths connecting a pair of neuronal somata. **(C)** *Pathway scale.* Each vertex represents a distinct cortical brain region in grey matter with a directed (or undirected) edge representing the axonal fiber tract connecting a pair of cortical regions, where its length describes the actual path or direct distance of its course within white matter.

To evaluate the proposed multi-scale wiring principle, we now examine the results from the analyses of graphical representations of cortical organization at three different spatial scales (see Figure 3): neuron, local circuit, and pathway scales. The reason for choosing these scales is that they offer a simple hierarchical organization of cerebral cortex into individual functional elements, connectivity within a cortical region, and connectivity between cortical regions, respectively. Local circuit (microscopic) and pathway (macroscopic) scales are represented in both approaches discussed in the previous section. But this scheme includes a neuron scale, which is absent in the Human Connectome proposal, because we are interested in how communication costs may have shaped neuronal morphology as well as cortical circuits. We recognize that this scheme limits our consideration to specific spatial scales and so may neglect subtle intermediate-scale wiring strategies.

### Neuron scale

To explain qualitative observations made from Golgi impregnated neurons, Ramón y Cajal proposed that neuronal morphology is controlled by laws separately conserving cellular material (“wire”), conduction delay (time), and brain volume (Cajal, 1899). Cajal did not attempt to quantify these relationships nor explain how they might interact. But he noted that in some cases the conservation laws might conflict “sacrificing economy of matter in favor of economy of time” (Cajal, 1899), which pre-dates observations made regarding cost trade-offs in network design (Section “Spatial Networks”).

To explain features of neuronal morphology and synaptic connectivity, research initially focussed almost exclusively on the role of conserving cellular material or wire length—the wiring minimization principle (Mitchison, 1991; Cherniak, 1994; Chklovskii et al., 2002). The ability of this solitary principle to explain connectivity has been highly influential in shaping thinking about brain design and explaining CNS connectivity (Chklovskii and Koulakov, 2004). For example, it was claimed that the wiring minimization principle could completely explain the wiring pattern of the roundworm *C. elegans* (Cherniak, 1994), the only fully mapped CNS connectome.

In recent years, however, a steady accumulation of evidence has eroded the over-riding importance of wire length minimization. In particular, the wiring pattern of *C. elegans* connectome is not strictly minimized for wire length because of the existence of long-range connections, which runs counter to the wiring minimization principle (Ahn et al., 2006; Kaiser and Hilgetag, 2006). The dominance of the wiring minimization principle, however, resulted in less attention being given to the other conservation laws of conduction delay and volume minimization and to understanding how these distinct laws interact. To redress the balance, two recent studies independently investigated Cajal's laws of material and conduction delay conservation in relation to the axonal and dendritic arbors of individual neocortical neurons, respectively.

#### Axon arbors

To analyze the wiring characteristics of single intracortical axon arbors, Budd et al. (2010) applied a range of graph theory optimization techniques to 19 *in vivo* reconstructions of excitatory spiny (pyramidal and spiny stellate) and inhibitory basket cells (Buzás et al., 2001; Kisvárday et al., 2002; Buzás et al., 2006). A 3D graphical representation was constructed for each axon arbor (see Figure 3A). Here, each fixed vertex corresponded to one of the thousands of putative presynaptic axonal boutons associated with the arbor plus a root vertex to represent the axon origin. The edges linking these vertices represented the arrangement of axon branches and collaterals in the arbor reconstructions. Edge weight was determined from the distance between the locations of connected vertex pairs. Total axonal wire length and average path length metrics were used to compare biological axon arbors against cost optimized artificial arbors.

**Figure 4:**
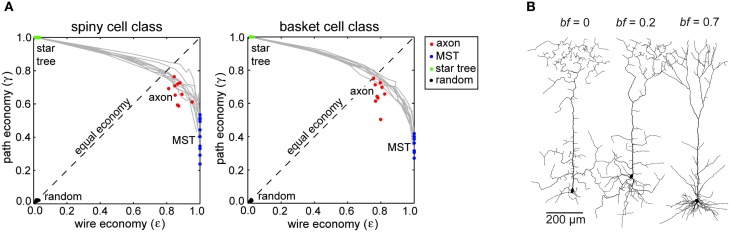
**Communication cost trade-off at Neuron scale of cortical organization. (A)** Similar degree of trade-off between path length and wire length economy of intracortical spiny (left) and basket cell axon arbors (right) between corresponding path length optimized star trees and wire length optimized minimal spanning trees (MST), which were all more economical than random arbors (Reprinted from Budd et al., 2010). **(B)** Examples of spiny pyramidal cell dendritic arbors generated using different trade-off balancing factor (*bf*) values show that the most realistic looking arbor was obtained for *bf* = 0.7 (Reprinted from Cuntz et al., 2010). Note *bf* parameter is equivalent to *β* parameter in Figure 2.

Neocortical axon arbors were not strictly minimized for either total axonal length or average path length; arbors used approximately 10–20% more axonal length than strictly necessary (Budd et al., 2010). Axon arbors used this excess wire to substantially improve average path length from axon origin to axonal boutons (Figure 4A). Strictly minimizing wire length only generated artificial arbors with a tortuous morphology and poorer average path lengths. In contrast, when artificial arbors were strictly minimized for path length only, they used a huge amount of axonal wire. Excess axonal wire in biological arbors was associated with branching close to the parent cell body and internodal axon segments lacking any boutons, which were often found between terminal branch clusters. Extrapolating from reported intracortical axon conduction velocity values (e.g., Luhmann et al., 1990), axonal path length distributions in this study suggested a narrow temporal dispersion of axonal latency within an arbor and a tight relationship between axonal latency and cortical distance (Budd et al., 2010). This prediction receives some support from the strong correlation between EPSP latency and cortical distance in the connections from layer 4 spiny neurons to layer 2/3 interneurons observed *in vitro* (see Figure 3C in Helmstaedter et al., 2008). Due to their greater branching complexity, the estimated axonal latencies of inhibitory basket cell were less dispersed than those of excitatory spiny cell axons. Thus, as expected for spatial networks generally (see “Spatial Networks”), neocortical axon arbors appear to trade-off communication costs using a small amount of extra axonal wire to ensure rapid and temporally precise signal propagation.

It should be mentioned that little is known about the trade-off across different species and neuronal types. The above study examined intracortical axons from adult cat primary visual cortex only and did not examine other neuronal types accounting for 10–15% of all neocortical neurons (see Budd et al., 2010). It would be interesting to know whether the communication cost trade-off extends universally to other neuronal types (typically short-axon types), cortical areas and species. In addition, axon diameter was not considered as an optimizing parameter in this study for reasons of computational complexity and measurement accuracy (see “Cortical Network Design Problem”). Finally, like other structural analyses, this study extrapolated temporal characteristics from published estimates of axonal conduction velocity. This general limitation of structural network analyses is examined in more detail later (see “Physiological Considerations”).

#### Dendritic arbors

To investigate how Cajal's principles might shape dendritic arbor morphology, Cuntz et al. (2010) “grew” artificial arbors starting from a single root vertex and then incrementally added edges to connect a set of sample (carrier) points. These sample points were selected uniformly at random from a 3D probability density distribution of branch and terminal points derived from multiple morphological reconstructions of actual pyramidal cell dendritic arbors in a specific cortical layer. At each iteration, a minimal spanning tree (MST) algorithm connected the existing arbor to the carrier point with the next lowest total cost until all points were connected to the tree (Cormen et al., 2001). Total cost was equal to the total wire length plus path length cost, which was multiplied by a balancing factor, *bf* (equivalent to β in Figure 2). For *bf* = 1, wire length and path length costs were treated equally; when *bf* = 0 then path length cost was ignored, minimizing wire length only. This approach has been previously applied to spatial network design problems outside neuroscience (Alpert et al., 1995; Gastner and Newman, 2006). Spatial jitter was randomly added to the artificial arbor vertices to mimic branch tortuosity in the neuropil. To estimate neuronal electrotonic cable properties, Cuntz et al. (2010) applied a radius-dependent dendritic diameter-tapering rule to the edges of the final artificial arbor to obtain the average electrotonic compartment size. Artificial arbors were morphologically compared with biological arbors using branch order and path length distributions and Sholl analysis descriptive statistics (Sholl, 1953).

Pyramidal cell dendritic arbors, regardless of cortical lamina, were best approximated by artificial arbors with a balancing factor of around 0.7 (Figure 4B). With a lower balancing factor, dendritic morphology was more tortuous and had a much greater average compartment size than observed in corresponding biological arbors. But Cuntz et al. (2010) noted pyramidal dendritic arbors had greater variability than dendritic trees in other neural structures. Interestingly, with a forest of growing arbors competing for carrier points, Cuntz et al. (2010) were able, by making each carrier point exclusive to the first arbor to which it became attached, to reproduce realistic individual artificial arbors and spatial tiling at the same time. These simulations demonstrate for the first time how balancing individual arbor communication costs may also conform to Cajal's law of brain volume conservation.

This study is, however, open to a number of criticisms. First, while artificial and biological pyramidal dendritic arbors visually appeared highly similar, no statistical tests for the degree of fit were reported. Second, to replicate the morphology of whole arbors the algorithm had to grow the apical tuft of an artificial neuron separately from its basal dendritic tree (see Cuntz et al., 2010). This suggests that the current algorithm might find it difficult to automatically reproduce more sparsely connected structures such as large axon arbors (e.g., Buzás et al., 2006). Third, the study was restricted to reproducing the morphological charcteristics of pyramidal cell dendritic trees neglecting those of other cortical neuronal types, notably aspiny or smooth type cortical neurons. Finally, it is not clear what biological mechanism could reproduce the results of the growth algorithm that appears to require global knowledge of vertex positions to compute total cost.

#### Arbor self-similarity

Relatedly, the morphology of neocortical axon and dendritic arbors have been separately described as possessing statistical self-similarity (e.g., Tettoni et al., 1998; Binzegger et al., 2005; Rothnie et al., 2006; Wen et al., 2009), implying common principles of arbor construction across a range of spatial scales.

To gain insight into the morphological diversity of afferent axon arbors innervating cerebral cortex, Tettoni et al. (1998) examined the metric and topological characteristics of 3D reconstructions of twenty-two callosal afferent axons from the area 17/18 border of cat visual cortex and seventeen thalamocortical afferent axons projecting from mouse ventrobasal thalamic nucleus to primary somatosensory cortex. Although these visually distinct axons derived from different species and represented different arbor types, they were similar metrically and topologically. Metrically, arbor types did not differ significantly in total axonal length, total number of branches, or branching angles. The topology of the two types of arbor proved highly similar when compared for maximal branching order (centripetal ordering scheme) and the distribution of branch order per arbor. However, arbor types were distinguishable in at least two respects. First, single thalamocortical arbors had on average five times more boutons with a higher proportion of branches with boutons than callosal arbors. Second, arbor types differed in their relationship between branch order and branch length: as branch order increased (distal to proximal direction), the individual branches of callosal axons tended to lengthen while thalamocortical axon branches shortened. Although there was individual arbor variability, this study suggests that corticocortical and corticothalamic afferent axons may share a common topology and differ only in a few metric parameters.

In a complementary study, Binzegger et al. (2005) systematically investigated the metric, topological, and fractal self-similarities of 3D reconstructions of spiny, smooth, and thalamic axon arbors intracellularly labeled in adult cat visual cortex (*n* = 39 axons). Here, each axon arbor was represented graphically with its edges corresponding to the axon's branch collaterals. Each edge was assigned a length corresponding to the length of the branch collateral and labeled to denote its topological order according to the Strahler centripetal scheme (see MacDonald, 1983). Arbor complexity was measured using the fractal box-counting dimension (see Addison, 1997). Binzegger et al. (2005) found that while smooth cell axon arbors branch more frequently than spiny cell axons (see also Budd et al., 2010) these arbors have similar branch length distributions. In addition, they reported the morphological diversity amongst arbor types masked a highly similar branching topology and statistical self-similarity (1.2–1.9 average fractal dimension). Smooth cell axons tended to exhibit greater complexity than either spiny or thalamocortical arbors. Although the majority of arbors showed statistical self-similarity, it remains unexplained why nearly 18% of arbors studied did not appear fractal-like. Together, the results of Tettoni et al. (1998) and Binzegger et al. (2005) imply a common principle of construction for thalamocortical and both extrinsic and intrinsic corticocortical axon arbors, where arbor topology is type-invariant but metric parameter values vary to alter axon branching patterns and synaptic bouton density.

In analyzing the basal dendritic arbors of pyramidal cells, Wen et al. (2009) reported evidence of a statistical self-similarity in the shape of 3D arbor reconstructions from cat visual cortex and a scaling correlation between arbor radius and dendritic length for these and more than two thousand 2D arbor images from primate neocortex. To explain these results, Wen et al. (2009) initially hypothesized that dendritic arbors sought only to maximize the number of different combinations of potential synapses, axon and dendritic appositions within a dendritic spine's length (Stepanyants and Chklovskii, 2005). Yet this unconstrained objective function (entropy maximization) generated space-filling artificial arbors with a tortuous morphology, because the branches sought both to maximize total arbor volume and spread out to avoid receiving multiple potential synapse from the same axon. When the objective function was constrained by path length cost (conduction delay), however, the less tortuous artificial arbor morphology was more realistic. This result highlights the importance of conduction delay conservation as a constraint on neuronal arbor design and communication.

In a bold unifying approach, Snider et al. (2010) proposed that all axonal and dendritic arbor types could be described by a single truncated Gaussian spatial density function (envelope of averaged arbor branching density). Yet a unimodal kernel cannot, for instance, properly portray the spatial clustering of axon terminals observed within the extent of long-range basket and pyramidal cell axon arbors (e.g., Kisvárday et al., 2002; Binzegger et al., 2005, 2007; Budd et al., 2010). Moreover, this approach had to describe separately the apical and basal dendritic trees of the same pyramidal neuron (Snider et al., 2010). Although Snider et al. (2010) acknowledged their approach was not directly concerned with branching topology, this work does emphasise the universality of dense arbor branching close to the parent cell body, identified as a source of excess wire length that helps reduce average axon path length (Budd et al., 2010).

#### Summary

Structural evidence for balanced communication costs in single cortical axons and dendritic arbors appears compelling, though it remains to be seen whether this wiring principle is universal across all neuronal types, cortical regions, and species. To test structural predictions, *in vivo* two-photon calcium imaging microscopy might be used to reconstruct the morphology of single cortical axon and dendritic arbors and then measure the latency of signal propagation at various arbor locations (e.g., Katona et al., 2011). However, as the field of view of two-photon microscopy is currently limited to less than 1 mm it would provide only a partial test for axon arbors (Katona et al., 2011).

Importantly, the results together suggest intracortical axon and dendritic arbors may well follow the same wiring principle. If so this principle has at least three advantages for efficient cortical design. First, it may provide the basis for arbor scaling because it will, unlike wire length minimization, allow for the addition of branches without significantly degrading communication. Second, time and distance in cerebral cortex will be strongly and positively correlated to promote temporal coherence between simultaneously excited but equidistant sources. Third, it implies a highly efficient genetic encoding of morphological neuronal differentiation may account for neuronal diversity though variation in the expression of relatively few molecular factors (see Dent et al., 2011). We next consider whether this wiring principle applies at larger spatial scales of cortical organization.

### Local circuit scale

Although individual axonal and dendritic arbors may separately trade-off structural communication costs, it cannot be assumed that these necessary conditions are together sufficient for local cortical circuits to also trade-off communication costs. When constructing graphical representations of local cortical circuits, edge lengths must combine both the axonal path length from presynaptic cell body and dendritic path length from synapse to postsynaptic cell body (see Figure 3B). This means that axosomatic or axoaxonic connections should tend to be less costly than axodendritic synapses, which are by far the most common variety of cortical synapse (Beaulieu and Colonnier, 1985; Schüz and Palm, 1989; Beaulieu et al., 1992). Somatic size limits the number of axosomatic connections to at most a few hundred (Fariñas and DeFelipe, 1991). Nonetheless these synaptic inputs probably have a robust influence on firing due to the closeness of their synapses to the action potential initiation zone, such as inhibitory basket cell axons and axoaxonic cell contacts regulating the phase of oscillatory firing (e.g., Cobb et al., 1995; Klausberger et al., 2003). On the other hand, the spatially extended dendritic arbors allow cortical neurons to each receive thousands of synaptic inputs (Feldman, 1984; Larkman, 1991). In deciding whether communication costs have been optimized in local cortical circuits, however, we need to know whether particular types of axon are constrained to target subcellular domains of dendritic arbors in cerebral cortex (see Somogyi et al., 1998). If so then a fixed offset should be subtracted from the edge length to compensate for this constraint. Yet the evidence that different types of axonal pathways innervate distinct subcellular domains of postsynaptic neurons is not clear-cut, e.g., thalamocortical axons (Ahmed et al., 1994; Bagnall et al., 2011 cf. da Costa and Martin, 2011).

Due to the lack of morphologically reconstructed local cortical circuits, there have to our knowledge been no published empirical studies of structural communication costs in cerebral cortex at this scale. Instead we will focus on what is known about the network topology of perhaps the most studied subcircuit of neocortex.

#### Layer 5 networks

To investigate the topology of local circuits, network analysis has been applied to data obtained from multiple simultaneous *in vitro* electrophysiological recordings of thick tufted layer 5 (TTL5) pyramidal cells taken from immature rat neocortical slice preparations (Song et al., 2005, postnatal day, P12–20; Perin et al., 2011, P14–16). Using differential infra-red microscopy, this cell type is readily identifiable for recording because of its relatively large cell body (Markram et al., 1997). Consequently, the electrophysiology, morphology, and synaptic properties of TTL5 neurons have been studied extensively *in vitro* (Chagnac-Amitai and Connors, 1989; Larkman, 1991; Markram et al., 1997). TTL5 pyramidal cell networks, which are sparsely interconnected via recurrent collaterals (~10%) (Markram et al., 1997), are of interest because they may be able to generate coherent theta-band oscillatory activity in neocortex (Chagnac-Amitai and Connors, 1989; Budd, 2005).

To investigate the degree of randomness in local cortical circuit connectivity, Song et al. (2005) analyzed quadruple whole-cell recordings of over 800 TTL5 pyramidal neurons in visual cortex obtained in a previous study (Sjöström et al., 2001). In these quadruple recordings, action potentials were evoked in each neuron in turn while recording the strength of any excitatory postsynaptic response in the remaining cells. Song et al. (2005) used these data to construct directed graphs for each quadruple recording group. They concentrated their analysis on three neuron groups for which there are 16 topologically distinct possible subgraph configurations. By generating random networks using their own estimates of unidirectional and reciprocal connection probabilities, Song et al. (2005) reported the existence of a number of three neuron motifs, subgraphs that occur more frequently than expected by chance, but only two of these achieved levels of statistical significance. Importantly, Song et al. (2005) found the more interconnected motifs tended to have stronger excitatory connections, from which they inferred a general network architecture consisting of a skeleton of strongly interconnected motifs surrounded by weaker and less connected neuronal motifs. They concluded the connectivity of TTL5 pyramidal cell networks were highly non-random.

Following a similar approach in somatosensory cortex, Perin et al. (2011) recorded from up to 12 pyramidal cells at a time for a total of over 1300 neurons. Like Song et al. (2005) they too discovered specific three- and, in addition, four-neuron motifs that were over-represented but obtained statistical significance only when recording groups contained six or more neurons. This confirmation of TTL5 pyramidal network structure suggests local cortical circuits may be composed of repeated elementary subnetworks, where each type of network motif serves a specific computational function (see Milo et al., 2002).

In addition, Perin et al. (2011) observed that the connection probability for a given neuron pair rose linearly with the number of connections they shared with other neurons—*“common neighbour”* rule. This relationship was stronger for shared input rather than output connections. This observation was independent of the intersomatic separation distance within the slice (up to 0.3 mm). Note the term “common” here does not necessarily imply neurons were spatially close. Perin et al. (2011) found neurons participating in a given motif which were often spread out spatially (100–125 μm).

Using the common neighbor rule and empirically based estimates of first-order distance-dependent connection probabilities, Perin et al. (2011) generated and analyzed an artificial network of 2000 point neurons. In this model, they identified nearly 40 spatially interlaced neuronal clusters, each of around 50 neurons. The model topology lacked the characteristics of scale-free, random, or regular networks but instead demonstrated small world clustering; the average shortest path length (unweighted) between any two neurons within a cluster was 1.9 hops, which is equivalent to 2.9 edges (synapses); hop count is the number of intermediate vertices (neural elements) that must be “hopped over” in a given path (see Box 1). This pattern of connectivity suggests a neuron may communicate more easily with others within a cluster regardless of its spatial location but less well with cells in other clusters even when they are spatially adjacent.

These two studies are significant because they hint at a local circuit topology that favors a high degree of clustering (small worldness) and emphasize the importance of higher-order statistics to determine connection probabilities. These higher-order statistics indicate that to understand local circuit connectivity requires more information than can be obtained from paired cell recordings.

Yet as neither study reconstructed and traced the processes connecting individual neurons, we cannot determine whether these local circuits were optimized for structural communication costs. We are unable to infer this, for instance, from electrode spatial locations, typically around 0.05 mm (e.g., Song et al., 2005), because these can these only give intersomatic distances between cell pairs. The intersomatic distance ignores the length of axon connecting a cell pair and the intracellular distance from dendritic synaptic input location to cell body, which alone is generally greater than the electrode separation distance (0.08–0.58 mm, Markram et al., 1997). To obtain the necessary data on whether the communication trade-off hypothesis holds at the local circuit scale of cerebral cortical organization, therefore, requires a reconstruction of the neuronal processes and tracing the connections of many individual neurons participating in a local cortical circuit—a formidable task for future studies.

#### Mapping morphology in local circuits

To help reconstruct local circuits, large-scale volume serial-section EM is now being used to map the fine structure of the cortical neuropil (e.g., Mishchenko et al., 2010; Bock et al., 2011). Manual annotation and tracing requirements of the very many EM images involved, however, severely limits progress. For example, Bock et al. (2011) examined a relatively small volume of mouse visual cortex (0.008 mm^3^), containing large portions of around 1500 upper layer neurons, but manually mapped only a tiny fraction of all synapses (~0.003%, 250 synapses out of 1 × 10^9^ total synapses per mm^3^ reported in Schüz and Palm, 1989). To completely survey even relatively small cortical volumes within a reasonable time scale, automated or semi-automated annotation, and tracing methods under development (e.g., Chothani et al., 2011; Helmstaedter et al., 2011) will need to improve productivity by many orders of magnitude without endangering quality. Mapping small cortical volumes though will not account for the significant fraction of intrinsic connections originating from outside the local neuropil volume (Stepanyants et al., 2009) without the use of additional labeling techniques. The productivity gains achieved in the history of Genome mapping, however, does give cause for optimism (see Lichtman and Sanes, 2008).

The current Human Connectome framework (Sporns et al., 2005) does not appear to explicitly incorporate morphological descriptions of neuronal processes. Yet to evaluate structural hypotheses at the local circuit level, morphological descriptions are required. The morphology of an axonal or dendritic arbor cannot be recovered from a connectivity matrix alone because of non-uniqueness. Specifically, for a neuron forming *N* synaptic connections, the connectivity matrix data could be accounted for by any one of *N*^(*N*−2)^ possible distinct tree configurations (Cayley's formula, Cormen et al., 2001). Reconstrucing a neuron's morphology is not only essential to estimate communication costs in cortical circuits but vital for understanding signal integration and distribution. In dendritic trees, for example, synaptic integration is shaped by arbor geometry, synaptic motifs, and the spatiotemporal pattern of dendritic stimulation (see Sjöström et al., 2008; Branco et al., 2010). On the positive side, however, incorporating an explicit 3D morphological description into the Human Connectome framework should not involve much additional computational cost. This is because to construct a connection matrix the paths of neuronal processes connected to a synapse must be traced back to the respective presynaptic and postsynaptic neurons (e.g., Mishchenko et al., 2010).

#### Summary

The absence of sufficient data for estimating structural communication costs at the local cortical circuit is a major hurdle in evaluating the multi-scale hypothesis. It is probable that soon fragments of a canonical cortical circuit will be reconstructed to permit the estimation of structural communication costs. In the meantime, progress in evaluating the hypothesis might be made by examining whether any trade-off between communications costs varies as the number of neurons per network motif increases.

### Pathway scale

Cortical regions are connected via fiber tracts, bundles of efferent axons of various calibers and mostly myelinated, that course through white matter to their target cortical region (see Salin and Bullier, 1995). On entering gray matter, each axon ramifies to produce one or more terminal arbors that synapse with many postsynaptic neuronal processes in a characteristic laminar-specific pattern (Salin and Bullier, 1995). Individual axonal pathways may be classified as feedforward (traveling away from primary sensory areas), feedback (traveling toward primary sensory areas) or lateral (Rockland and Pandya, 1979; Felleman and Van Essen, 1991; Rockland, 1997). Based on the differential laminar termination patterns and physiological effect of afferent axons, feedforward pathways are thought to drive neuronal activity while feedback pathways act to modulate neuronal gain (Johnson and Burkhalter, 1996, 1997; Budd, 1998; Larkum et al., 2004; Rothman et al., 2009). Macroscopic cortico-cortical networks may be constructed by mapping fiber tracts to their end points to define regional vertex positions and using the tract itself to define the connecting edge (see Figure 3C). Edge costs can be defined according to tract morphological properties such as fiber length and fiber density/number.

#### Tracer-derived networks

**Figure 5:**
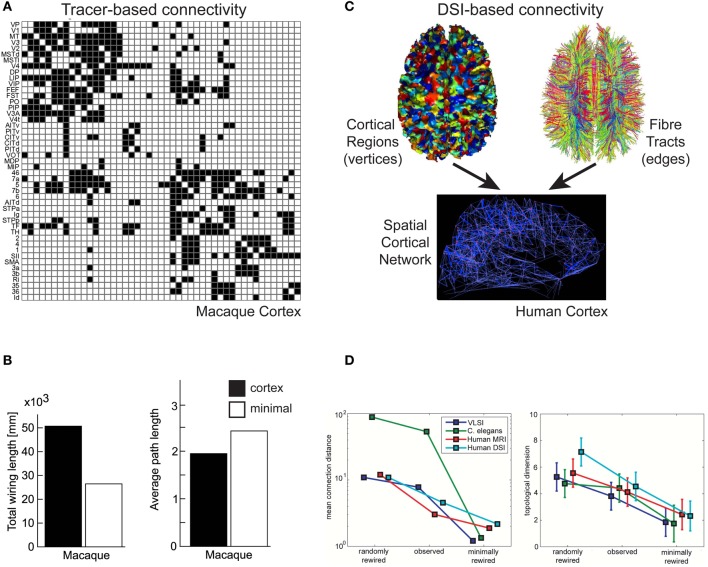
**Communication cost trade-off at Pathway scale of cortical organization. (A,B)** Macaque tracer-derived pathway connectivity. **(A)** Example of directed connectivity (adjacency) matrix of visual and somatomotor macaque cerebral cortex (Reprinted from Sporns et al., 2007), where black squares indicate evidence supporting a axonal pathway connection between areas (matrix rows as sources and columns as target cortical regions). **(B)** Macaque cerebral cortical network is suboptimal for total axonal length (left) but minimal length network increased averaged path length (right) (Reprinted from Kaiser and Hilgetag, 2006). **(C,D)** Human DSI-derived pathway connectivity. **(C)** An undirected spatial cortical network (bottom) is constructed from vertices of cortical regions (top, left) and edges determined from the probability of fiber tracts existing between corresponding pairs of cortical regions based on tractography tracing algorithms (top, right) (Reprinted from Hagmann et al., 2008). **(D)** Human DSI network is suboptimal for wire length (left) but minimal length network has lower topological dimension than observed cortical network (right) (Reprinted and partly redrawn from Bassett et al., 2010). Topological dimension here is a fractal measure of a network's degree of internal connectedness.

By injecting molecular labeling agents such as biocytin or *Phaseolus vulgaris*-leucoagglutinin (PHA-L) into anatomically identified cortical regions, the origin or termination sites of individual cortical axons can be traced over long-distances (Rockland, 2004). Individual axons can have quite convoluted trajectories (Rockland, 1997) and may diverge to terminate in more than one cortical area (Schwartz and Goldman-Rakic, 1982; Bullier et al., 1984). By combining results from different laboratories for a variety of tracer agents, it has been possible to construct draft qualitative adjacency matrices of inter-areal cortical connectivity (Felleman and Van Essen, 1991; Scannell et al., 1995), of which some are publicly available, e.g., CoCoMac database for macaque cerebral cortex (http://cocomac.g-node.org/drupal/, Stephan et al., 2001; Kötter, 2004). An example is shown in Figure 5A. Analyses of these draft connectivity matrices have proved useful in, for example, establishing the high incidence of reciprocal pathways in macaque cortex (Felleman and Van Essen, 1991), offering an anatomical basis for separate ventral and dorsal functional streams in visual cortex (Young, 1992), and discovering “nearest-neighbour” and “next-nearest-neighbour” connectivity rules between cortical areas (Felleman and Van Essen, 1991; Young, 1992). But in the latter case these rules only partly explain the full connectivity matrix (Scannell et al., 1995; Costa et al., 2007).

To discover whether cerebral cortex macroscopic networks were minimized for wire length, Kaiser and Hilgetag (2006) used CoCoMac data to construct a 3D spatial network. This network comprised 95 vertices, where each vertex represented the 3D centre-of-gravity of a distinct cortical area or subarea obtained from a standardized cortical parcellation surface map, and 2402 edges, where each edge represented a pathway revealed by tracer injections. Edge cost was estimated from the distance between the 3D positions of vertex pairs rather than the actual length of the fiber tract. Kaiser and Hilgetag (2006) found the macaque cortico-cortical network does not appear to be strictly minimized for wire length. Optimization algorithms were able to significantly shorten the total wire length of the original macaque network (>30%) mainly by reducing the number of long-distance connections (Kaiser and Hilgetag, 2006), though this significantly increased the average path length (hops) between cortical areas (Figure 5B). Kaiser and Hilgetag (2006) concluded that the excess wire associated with long-range pathways introduced shortcuts to reduce the number of “processing steps” between cortical areas. This conclusion is consistent with the results from the optimization analysis of single intrinsic axon and dendritic arbors (Budd et al., 2010; Cuntz et al., 2010).

Does it matter that this study assumed straight-line fiber trajectories? Kaiser and Hilgetag (2006) acknowledged their assumption underestimated pathway length, though in fairness, there is insufficient actual fiber length data available. This issue may not be a significant problem provided tract curvature is relatively constant, i.e., if pathways are all similarly curved. In primate prefrontal cortex, for example, around 55% of the fiber tract trajectories measured were approximately straight though the remainder had some degree of curvature with denser tracts tending to be straighter than sparse ones (Hilgetag and Barbas, 2006). Moreover, these measurements are not easy to make because initially compact fibers bundles can splay and divide so that fibers may take different trajectories through white matter (Hilgetag and Barbas, 2006). This uncertainty about the effect of tract length on communication costs makes it important to investigate this issue further.

In general, network analysis based on tracer study data has been impeded by the absence of a systematic quantification of axonal pathways properties such as axon length or axon (connection) density (see Salin and Bullier, 1995). The CoCoMac database, for example, provides a limited integer rating of connection density/strength but no information on pathway length (Stephan et al., 2001; Sporns et al., 2007). There are understandable reasons for the absence of these data. Tracer label may not fill all axonal branches especially when it is of a fine caliber (<1 μm) and plotting the trajectory of long, fine cortical axons, and measuring their structural parameters is hugely time-consuming (Salin and Bullier, 1995; Rockland, 2002). Moreover, tracer studies report considerable between-individual variability in the fiber density of specific cortio-cortical pathways (e.g., Scannell et al., 2000). Even when the labeling methodology is carefully controlled, Markov et al. (2011) found, as well as discovering previously unreported pathways to well-studied visual cortical areas, that connection density could vary upto five-fold between individuals. But caution should be used in assuming that pathways of equal axon number have an equal postsynaptic effect on target neurons, especially when one may be feedforward and the other feedback (Johnson and Burkhalter, 1997). Moreover, connectivity matrices derived from the composite results of tracer injection studies tacitly assume that network topology is the same for all individuals of the same species. It is unclear whether this assumption is secure.

#### MRI-derived networks

Axonal tracer studies can only be used map relatively few fiber tracts per animal (see Salin and Bullier, 1995) making them ill-suited to map all extrinsic cortico-cortical pathways in an individual brain. Recently, by combining two complementary *in vivo* MRI techniques whole macroscopic cortico-cortical networks can be mapped non-invasively for an individual brain. An example is shown in Figure 5C. This approach offers the possibility to study individual differences in health and disease (Bassett and Bullmore, 2009; Hagmann et al., 2010).

In the first stage, structural MRI is used to construct a 3D surface model of the cerebral hemispheres at the boundary where fibers enter and leave cortical gray matter. A standardized parcellation template is applied to the surface model to identify cortical areas in each subject, after which these areas are subdivided into distinct, equally sized regions of interest (ROI), typically ~cm^2^ of surface area (Hagmann et al., 2008; Echtermeyer et al., 2011). Each network vertex corresponds to a distinct ROI (Figure 5C, top, left).

In the second stage, to help construct network topology, diffusion MRI (diffusion spectrum imaging, DSI) is used to trace fiber tracts to and from ROIs. Fiber tracts are identified and traced from the anisotropic diffusion of water molecules along their length (Moseley et al., 1990; Conturo et al., 1999; Wedeen et al., 2012). Tractography algorithms trace paths of maximal diffusion coherence (correlation) to generate a connection probability of each pseudo-fiber (see Hagmann et al., 2010; Figure 5C, top, right). Tract density and length may also be estimated from pseduo-fiber constructions (Hagmann et al., 2008), though fibers cannot be traced once they enter gray matter with this method because of the relative lack of anisotropic water diffusion here (Conturo et al., 1999). To decide whether to add an undirected edge to the network, a threshold is typically applied to the raw connection probability matrix (see Rubinov and Sporns, 2010; Figure 5C, bottom). Validation of DSI tractography for human cortex using tracer methods is not possible for obvious ethical reasons and dissection approaches are considered unreliable at this level of detail (Hagmann et al., 2010). However, the postmortem application of tract tracing histological tools (e.g., lipophilic tracer, DiI, Galuske et al., 2000) can be envisaged. Recently, Axer et al. (2011) have shown 3D-polarized light imaging applied to postmortem tissue can trace fiber tracts in white matter at a sub-millimeter resolution though it cannot yet follow individual fine caliber (<1 μm) axons.

To date, two studies have investigated the communication costs in human MRI-derived large-scale cortical networks (Hagmann et al., 2008; Bassett et al., 2010). Analyses of networks constructed from functional MRI data report similar results (e.g., Achard and Bullmore, 2007) though are not discussed here.

To non-invasively map inter-regional connectivity in human cerebral cortex, Hagmann et al. (2008) constructed and analyzed networks each of 998 ROI vertices for five healthy human subjects. An edge was inserted if at least one pseudo-fiber identified by DSI connected an ROI pair. In some versions of the network, edges were weighted with a length, l(e), based on the average length of pseudo-fiber trajectories, and a density, w(e), based on the number of pseudo-fibers per mm^2^. From their analysis, Hagmann et al. (2008) identified a network core consisting of a relatively small number of highly interconnected cortical hubs. By virtue of having low average path lengths to all other cortical regions, the network had high local efficiency. Six main modules were related to these hubs. The study reported a good but imperfect correspondence between the gross cortical pathways of macaque and human brains. The study did not, however, investigate whether network communication costs were optimized.

To investigate topology and wiring costs in human cortical macroscopic networks, Bassett et al. (2010) analyzed a modified version of the Hagmann et al. (2008) DSI network for single human subjects and, for comparison, a network derived on gray matter volumes covariation (GMC). GMC is an indirect anatomical marker for connectivity (He et al., 2007) obtained from averaging over many human subjects. In contrast to Hagmann et al. (2008), network topology was determined by thresholding connection probabilities between ROI pairs. Moreover, while edges were assigned a length cost, based on the distance between ROI pairs instead of pseudo-fiber length, they were not given a density parameter. Bassett et al. (2010) acknowledged this is likely to underestimates actual fiber length but they judged pseudo-fiber measurements unreliable (see later). Bassett et al. (2010) reported evidence of network modularity over a range of scales, consistent with statistically self-similar connectivity. This hierarchical modularity or “modules within modules” architecture (Simon, 1962) was more clearly defined in DSI than GMC networks. The “modules within module” architecture offers a high degree of network efficiency (see “Spatial Networks”) because in general fewer vertices are traversed to reach a target vertex than in regular or random architectures (Simon, 1962). Importantly, Bassett et al. (2010) found human cortical networks were not strictly minimized for wire length (Figure 5D, left). Indeed, strictly minimizing wire length reduced or eliminated hierarchical modularity, which in turn reduced the cost-efficiency of balancing topological complexity within available physical space (Bassett et al., 2010; Figure 5D, right). Topological complexity here was reported using a fractal measure of the network's topological dimension, its degree of internal connectedness. These findings agree with the conclusions of Kaiser and Hilgetag (2006) regarding the balancing of communication costs in macroscopic networks.

Networks constructed using DSI are, however, subject to limitations. First, diffusional MRI fiber tracing suffers from a distance bias. Long-range fibers may not be reliably traced because of an exponential decrease in coherent diffusion with distance and short tracts may not be detectable due to the spatial sampling limitations of MRI (Hagmann et al., 2007; Gigandet et al., 2008). Second, tractography algorithms have difficulties segmenting proximal fiber tracts such as distinguishing “kissing” from crossing fiber bundles because MRI lacks the spatial detail of tracer studies, which may also mean that splaying fiber tracts are under-represented (Schmahmann et al., 2007; Gigandet et al., 2008; Hagmann et al., 2010). *In vivo* MRI spatial resolution is limited to at best a few hundred microns (e.g., Schmahmann et al., 2007). Third, ROI size choice affects network topology (Hagmann et al., 2007; Echtermeyer et al., 2011) with implications for drawing robust inferences about network properties such as motif distributions (Echtermeyer et al., 2011). Fourth, unlike tracer studies, diffusional MRI cannot provide information concerning the laminar origin or termination of tract axons in gray matter (Conturo et al., 1999). Hence, MRI-derived networks cannot currently employ directed edges. The use of undirected edges, however, tacitly assume that communication has an equal (postsynaptic) impact in either direction when, as mentioned earlier, there are reasons for believing this may not be the case, e.g., feedforward vs. feedback cortical afferent pathways (Rockland and Pandya, 1979; Felleman and Van Essen, 1991; Johnson and Burkhalter, 1996, 1997; Rockland, 1997; Budd, 1998; Larkum et al., 2004; Rothman et al., 2009). In addition, while in primate cerebral cortex most axonal pathways between cortical areas are reciprocal it appears some are not (Felleman and Van Essen, 1991). Finally, thresholding connection probabilities to determine whether an edge exists necessarily generates different network topologies for the same dataset: high thresholds lead to sparsely connected networks that may accidentally eliminate weak but actual pathways, while low thresholds may result in densely connected networks including erroneous edges generated by noise.

#### Summary

Evidence from two studies, one based on a composite network of axonal tracer data of macaque cortex (Kaiser and Hilgetag, 2006) and the other from individual MRI-based human cortico-cortical networks (Bassett et al., 2010), offer support for the main hypothesis. Both studies concluded that, like single intracortical axonal (Budd et al., 2010) and dendritic arbors (Cuntz et al., 2010), cortical wiring is not strictly length-minimized with excess wire used to promote rapid communication. To better understand corticocortical communication costs at this scale, however, future research will need to incorporate the density of connection and postsynaptic effect of fiber tracts into network models. Given such a wide range of individual differences in connection density (e.g., Scannell et al., 1995; Markov et al., 2011), it seems improbable that this variation has a negligible influence on information flow.

## Physiological considerations

**Figure 6:**
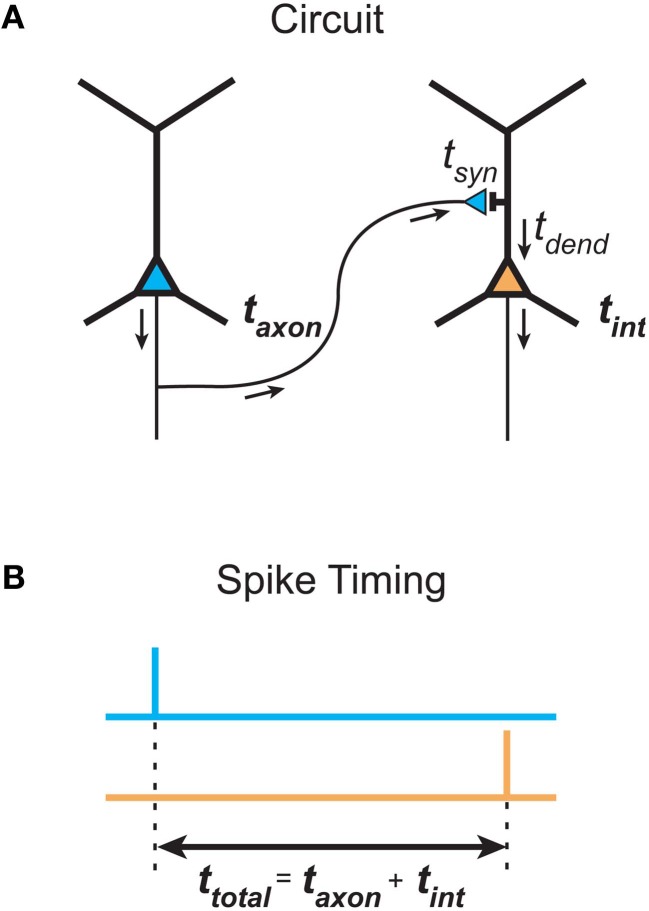
**Total communication delay between neurons separable into a presynaptic axonal conduction delay and a postsynaptic neuronal integration delay. (A)** Schematic circuit diagram shows the time taken by an action potential generated in the presynaptic neuron (left neuron, blue) to propagate along the axon to a presynaptic terminal, where it causes the release of neurotransmitter into the synaptic cleft, defines the axonal conduction delay (*t*_axon_). The postsynaptic neuronal integration delay (*t*_int_) is the sum of the time taken for neurotransmitter molecules to induce a local postsynaptic response (synaptic delay, *t*_syn_) and the latency for this response to propagate down the dendritic tree to the axon initial segment, where its integration produces an action potential in the postsynaptic neuron (right neuron, orange) (dendritic delay, *t*_dend_). **(B)** Total delay (*t*_total_) between the timing of a presynaptic spike occuring (top, blue line) and the generation of a postsynaptic spike (bottom, orange line) is determined by the sum of presynaptic and postsynaptic delay components.

What is the justification for inferring temporal cost (delay) from path length in spatial networks? The total signaling delay of a stimulus in a cortical network, *t*_total_, may be separated into two main components (Nowak and Bullier, 1997): a presynaptic component, axonal conduction delay, and a postsynaptic component, neuronal integration delay (see Figure 6). We now examine these distinct components and consider how they should be used in estimating temporal cost in cortical networks at different spatial scales.

### Presynaptic temporal cost

Presynaptic axonal conduction delay (*t*_axon_) is the time taken for an action potential to propagate from its initiation site at the axon initial segment (AIS) or axon hillock (Stuart et al., 1997) along the axon arbor to a given presynaptic terminal (see Figure 6A).

Axonal conduction delay may be estimated from the product of the weighted path length between axon origin and presynaptic terminal and the average conduction velocity along this path (see *L*_axon_, Figure 3B). Weighted path length is thought to be the major determinant of conduction delay for intracortical axon arbors (Manor et al., 1991). Conduction velocity increases with axon diameter and in the presence of myelination but may be reduced when branch points and axon varicosities are encountered (see Debanne et al., 2011). Intrinsic corticocortical axons have a narrow, positively skewed diameter distribution (0.1–1.0 μm) with the majority of their arbor length composed of unmyelinated branches, as so far reported (e.g., Haug, 1968; Braitenberg and Schüz, 1991; Peters and Sethares, 1996). In contrast, extrinsic corticocortical axons, though with a similarly shaped diameter distribution, tend to be thicker (e.g., ~1–3 μm in macaque) and most are myelinated along their length until arborization (e.g., Houzel et al., 1994; Anderson and Martin, 2002; Wang et al., 2008). Correspondingly, the conduction velocity of intracortical axons is generally reported as slower (0.1–0.6 m/s, Komatsu et al., 1988; Luhmann et al., 1990; Hirsch and Gilbert, 1991; Lohmann and Rörig, 1994; Feldmeyer et al., 2002) than extrinsic corticocortical pathway axons (>1 m/s, Harvey, 1980; Girard et al., 2001). Typically axonal pathways are heterogeneous, composed of axons with a range of calibers (e.g., Anderson and Martin, 2002; Wang et al., 2008) and conduction velocities (Harvey, 1980; Girard et al., 2001). Interestingly, conduction latency distributions between reciprocally connected cortical areas overlap (Raiguel et al., 1989; Girard et al., 2001). In general, action potentials are reliably transmitted throughout cortical axon arbors (Cox et al., 2000) even along thin varicose branches at least up to 100 Hz (Raastad and Shepherd, 2003). However, propagation failures can occur under certain circumstances such as spike bursting (Raastad and Shepherd, 2003; see Debanne et al., 2011). Short-term spiking history can induce a modest change in conduction velocity in some types of cortical axon (2–22%, Swadlow et al., 1978; Shlosberg et al., 2008). Overall, these findings suggest that when mean conduction velocity is known it is not unreasonable to estimate presynaptic axonal conduction delay from weighted path length.

### Postsynaptic temporal cost

Postsynaptic neuronal integration delay (*t*_int_) is the time taken for postsynaptic depolarization arising from a given presynaptic axon to generate one or more action potentials in response (see Figure 6A). We divide this delay into two subcomponents.

First, there is a synaptic delay (*t*_syn_), the time taken for neurotransmitter molecules released presynaptically to activate postsynaptic receptors, which for glutamate and GABA fast transmission at central synapses is thought to be brief (<0.5 msec, Sabatini and Regehr, 1996; Markram et al., 1997). The amplitude and width of the presynaptic action potential, however, can affect the degree of synaptic delay (Boudkkazi et al., 2007, 2011).

Second, there is a dendritic delay (*t*_dend_), the time taken for the local postsynaptic dendritic depolarization to induce one or more action potentials following its propagation to and integration at the AIS. The dendritic propagation delay depends on dendritic path length (*L*_dend_, see Figure 3B) and the mean dendritic conduction velocity along this path. Dendritic conduction velocity depends on the electrical properties of these dendritic branches, which is in turn influenced by arbor geometry such as branching ratio and dendritic diameter and, importantly, whether the signal is conducted actively or passively along the dendritic branch (see London and Häusser, 2005; Spruston, 2008). Morphologically, a pyramidal cell dendritic arbor, for example, is typically composed of a largely spherical basal dendritic arbor around the cell body and a main apical dendritic trunk, oriented toward the pia, which emits a number of proximal oblique branches before bifurcating to produce a densely branched distal apical tuft (Feldman, 1984). Passively conducting EPSPs in the basal and oblique apical branches conduct rapidly to the soma (at most a few milliseconds) but EPSPs from distal apical tuft can take longer (up to 10 msec or more) (Agmon-Snir and Segev, 1993; Markram et al., 1997; Ulrich and Stricker, 2011). The synaptic activation of voltage- and calcium-dependent dendritic spiking amplifies and more rapidly conducts dendritic EPSPs to the soma from all locations of the pyramidal dendritic tree (Yuste et al., 1994; Larkum et al., 1999; Schiller et al., 2000; Nevian et al., 2007; Larkum et al., 2009). *In vivo*-like spontaneous synaptic background activity, observed in neuronal recordings of awake animals, differentially reduces the delay of distal compared with proximal dendritic EPSPs (Rudolph and Destexhe, 2003a). The higher conductance state decreases the effective membrane time constant that regulates the rate of temporal integration, so the neuron responds more readily to sharp fluctuations in membrane conductance than slowly changing dendritic signals (Rudolph and Destexhe, 2003b). However, intrinsic delayed potassium currents in cortical neurons may defer spiking (Storm, 1988; Beggs et al., 2000). Hence, there is more scope for variability in the postsynaptic than the presynaptic component of total signaling delay.

Based on *in vivo* electrophysiological recordings following sensory stimulation, Nowak and Bullier (1997) estimated that the minimum neuronal integration delay for quiescent (low conductance state) cortical neurons was 5–10 ms but faster at 1–5 ms for already depolarized neurons (high conductance state). This suggests the level of spontaneous synaptic background activity may regulate neuronal integration delay. Based mostly on cat and monkey data, the minimum total signaling delay between cortical areas is thought to be around 10 msec (see Nowak and Bullier, 1997), though it might be different in other species such as rodents.

### Estimating temporal cost in networks

Figure 1 illustrated that the shortest path length between a given pair of vertices in the same network may be different depending on whether path length was measured using the number of edges/hops (unweighted path length) or the sum of edge lengths (weighted path length). While weighted path length only was used to estimate temporal cost in intrinsic axonal and dendritic cortical arbors (Budd et al., 2010; Cuntz et al., 2010), both measures have been used for local cortical circuits and large-scale extrinsic cortico-cortical networks (Kaiser and Hilgetag, 2006; Bassett et al., 2010; Perin et al., 2011).

So which path length measure is the most appropriate to use to estimate temporal cost at each spatial scale of cortical organization? At the single neuron scale, temporal cost is isolated from the network in which it is embedded. Hence, temporal costs estimated from either axonal or dendritic weighted path length can assume average levels of activity. Yet for local or large-scale cortical networks scales we are interested in combined presynaptic and postsynaptic delays, which will vary according to the conductance state of each neuron traversed in a path.

There are three main parameter regimes to consider here. First, when presynaptic axonal conduction delays are much longer than the postsynaptic neuronal integration delays (*t*_axon_ >> *t*_int_) then weighted path length dominates total signaling delay estimates. This regime operates when, relative to the other source of delay, axons are long or integration delays brief. Second, when presynaptic conduction delays are much shorter than postsynaptic neuronal integration delays (*t*_axon_ << *t*_int_) then hop count becomes a more representative measure of total signaling delay. This regime occurs when axons are relatively short or integration delays long. Third, when the presynaptic conduction delay is of a similar order of magnitude to the postsynaptic neuronal integration delay (*t*_axon_ ~ *t*_int_) then a combined measure should be used to estimate total signaling delay.

To examine under which parameter regime local and circuit macroscopic pathway scale networks may operate, we calculated what percentage of estimated axonal delays (presynaptic component) fell within an order of magnitude of the minimum neuronal integration delay (postsynaptic component) (1–5 ms for high- and 5–10 ms for low-conductance or quiescent states; see Nowak and Bullier, 1997). We estimated axonal conduction delays based on empirical distributions of path length in individual spiny neuron axons from cat visual cortex (*n* = 22,001 paths, Budd et al., 2010) and fiber tract lengths estimated for macaque cerebral cortex (Kötter, 2004; Kaiser and Hilgetag, 2006; *n* = 2390 tracts; see www.biological-networks.org). To estimate axonal conduction delay, intrinsic spiny axon path lengths were divided by a realistic range of mean conduction velocities for intrinsic cortical axons (0.1–0.6 m/s, Komatsu et al., 1988; Luhmann et al., 1990; Hirsch and Gilbert, 1991; Lohmann and Rörig, 1994; Feldmeyer et al., 2002), while extrinsic fiber tract lengths were divided by a realistic range of mean conduction velocities for extrinsic cortical axons (1–10 m/s, Miller, 1975; Swadlow et al., 1978; Harvey, 1980; Girard et al., 2001).

**Figure 7:**
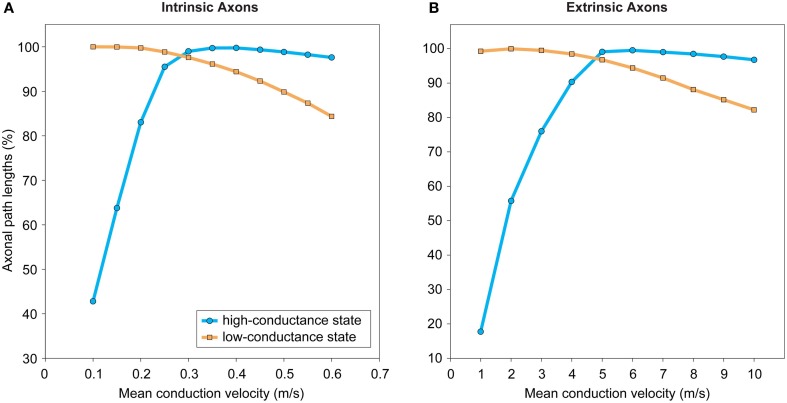
**Most estimated intrinsic and extrinsic axonal conduction delays are within an order of magnitude of neuronal integration delays apart from when axons conduct at their slowest rate and neurons operate in a high-conductance state. (A)** Intrinsic axon path lengths of spiny neuron within gray matter in adult cat cerebral cortex (*n* = 22,001 paths from 19 neuron reconstructions). Data taken from Budd et al. (2010). **(B)** Extrinsic axonal fiber tract lengths in adult macaque cerebral cortex (*n* = 2309 pathways). Data taken from Kaiser and Hilgetag (2006).

Figure 7 shows the results for both single intrinsic axon and extrinsic fiber tract data were quite similar. For the low-conductance state, virtually all axonal conduction delays were within an order of magnitude of neuronal integration delay almost regardless of mean conduction velocity (Figures 7A,B, square symbols). For the high-conductance state, except at the very lowest conduction velocities, the majority of the conduction delays were comparable to integration delays (Figures 7A,B, circle symbols). These results suggest that in both local and macroscopic cortical networks presynaptic axonal conduction delays may be mostly of a similar order of magnitude as postsynaptic neuronal integration delays (i.e., *t*_axon_ ~ *t*_int_). To determine the shortest path length between a pair of neural elements, therefore, it is important to take into account both the number of neural elements in the path as well as its physical length estimated from measuring axonal and/or dendritic processes. It is unclear whether assigning a cost for each vertex as well as each edge would significantly affect the results for cortical networks previously analyzed (Kaiser and Hilgetag, 2006; Bassett et al., 2010).

## Discussion

### Overview

Although cerebral cortex appears a jungle of axonal and dendritic wiring, as we explore deeper into its structure we find an order to its organization that helps explain how, in a vast network composed of billions of highly interconnected yet spatially distributed neurons, information is processed accurately and rapidly. In this article, we critically examined an hypothesis to help at least partially explain cortical wiring and connectivity at multiple scales of organization in terms of a trade-off between spatial and temporal communication costs (Budd et al., 2010). This hypothesis is grounded in Cajal's laws of conservation for cellular material and conduction delay (Cajal, 1899). We found supporting evidence for the hypothesis from studies applying network analysis at single neuron and macroscopic pathway network spatial scales. But a lack of available structural data prevented any meaningful evaluation at the local circuit scale. To progress this evaluation, we have identified the need for a more detailed morphological component in the Human Connectome framework. Recent advances in high-resolution cortical connectivity mapping (Mishchenko et al., 2010; Bock et al., 2011) make it timely to consider how morphological data should be recovered and suitably databased to aid analysis and modeling (e.g., Ascoli, 2007). To better estimate temporal cost in local circuit and macroscopic (pathway) scale networks, we suggest combining edge (axonal conduction) and vertex (neuronal integration) delays.

### Limitations of graph theory

Network descriptions, like other types of model, provide a simplified representation of a real world system. Yet there is a risk that viewing cerebral cortex simply in terms of discretely interconnected neural elements may blinker us to what Cajal (1937) referred to as “the utmost subtleties” of its structure. To gain insight into a phenomenon, the process of abstraction necessarily involves discarding some details considered less important though this risks leaving out key elements to its understanding. We now consider the appropriateness of applying graph theory to understanding cortical wiring.

Here, the abstraction of neural architecture into vertices (neural elements) and edges (neural connections) is most straightforward when there is a direct physical correspondence with distinct anatomical features. At the single neuron level, axon and dendritic arbors are easily identified from the visualized processes of neurons. In contrast, defining precisely what a vertex represents at the mesoscopic scale has proved problematic. The mesoscopic appears inextricably linked with the cortical column concept (Horton and Adams, 2005; Rockland, 2010; da Costa and Martin, 2010), where considerable controversy exists regarding its spatio-functional dimensions. While pathways may be easily identified, there is, however, no universally accepted scheme regarding how to divide cerebral cortex into regions (Van Essen et al., 2012a,b), which means there is no standard vertex set. Moreover, edge definitions can be more complicated at this scale. Modeling a reciprocally connected pair of cortical regions by a single edge (Hagmann et al., 2008; Bassett et al., 2010) ignores differences that may exist between feedforward and feedback pathways in the laminar termination pattern of their afferent axons and likely postsynaptic effect (Johnson and Burkhalter, 1996, 1997; Rockland, 1997; Budd, 1998; Larkum et al., 2004; Rothman et al., 2009). Ideally, each of pair of pathways should each be represented by a pair of directed edges and weighted in some way to record their relative influence on the flow of information. This is problematic for human cerebral cortex where these data are wholly absent. In addition, there is a tacit assumption that a single edge represents a fairly homogeneous fiber system whereas, like callosal pathways, it may be composed of a diverse range of myelinated and unmyelinated axons of various calibers (Houzel et al., 1994; Wang et al., 2008; Caminiti et al., 2009). A heterogeneous pathway may reflect the existence of a number of parallel functional streams that selectively target separate neuronal groups and/or at different conduction velocities, e.g., the pathway between area V1 to V2 in macaque monkey visual cortex (e.g., Sincich et al., 2010). To represent heterogeneous pathways, therefore, it might be more accurate to use multiple edges connecting a pair of distinct cortical regions, each assigned different properties. Finally, other forms of communication should not be forgotten such as local ephaptic interactions between neigboring neurons (Anastassiou et al., 2011) and cortical inhibition without direct synaptic connections (Oláh et al., 2009), as well as global extrasynaptic neuromodulation that can alter neuronal state (see Bargmann, 2012).

Despite these limitations, this review has provided ample evidence of the utility of graph theory abstractions to help gain insight into cortical design and concomitantly wiring economy. It should be borne in the mind that the success of such models depends not on their fidelity in replicating the physical features of the biological system but on the accuracy of their predictions and what insight this offers into the system studied.

### Cortical network design problem

Natural selection as a designing agent is a unifying concept in biology (Maynard Smith, 1978). It follows from this notion that the characteristics of brain architecture and function have adapted to improve an organism's survival in its environment (e.g., Kaas, 1989). Here, a cost function can be viewed as an hypothesis about what selective forces are responsible for cortical network design. A test of this hypothesis is how well cost optimization explains the characteristics of brain structure and function. In this article, we have focussed on two known costs concerning the cortical network design problem but clearly there are others. We now evaluate how well the wire length metric approximates spatial cost (cellular material) and consider the influence of other costs on cortical network design. Path length as an approximation for temporal cost was evaluated earlier (see “Physiological Considerations”).

Almost all optimization studies discussed in this review have approximated Cajal's conservation of cellular material to minimizing wire length (Mitchison, 1991; Cherniak, 1994; Koulakov and Chklovskii, 2001; Kaiser and Hilgetag, 2006; Wen et al., 2009; Bassett et al., 2010; Budd et al., 2010). The main assumptions underlying this approach are: (1) wire length is directly proportional to the amount of cellular material used; and, (2) distance traveled is directly proportional of the degree of conduction delay (Cajal, 1899; see Chklovskii and Koulakov, 2004). Although both assumptions are valid, this approach does not take account of other characteristics of neuronal processes that have a bearing on cellular material and conduction delay conservation, in particular axonal and dendritic diameter, which we now discuss.

Axonal and dendritic diameter regulates the rate of ionic diffusion per unit length responsible for the conduction velocity of electrochemical signals (see Debanne et al., 2011). Doubling the diameter of a myelinated axon, for instance, would be expected to halve the conduction time for a given length of axon because of the approximately linear relationship between axon diameter and conduction velocity (Hursh, 1939; Waxman and Bennett, 1972). However, for an unmyelinated axon the conduction time differential would be less because conduction velocity is proportional to the square root of axon diameter (Rushton, 1951; Hodgkin, 1954). Hence, an increase in axon or dendritic diameter causes a squared expansion in the volume of cellular material while conduction velocity increases, at best, linearly.

Currently, a lack of data prevents an optimization analysis combining axon diameter and length. Because of the spatial resolution limits of light microscopy (LM), EM is needed to accurately measure the finest caliber axons found in both extrinsic and intrinsic corticocortical axon pathways (see Peters et al., 1991). To control for morphological irregularities such as swellings, multiple sample points are needed to obtain the average diameter of an axon branch. Hence, for a single axon arbor hundreds of diameter measurements under EM might be required. Population axon diameter distribution data does exist but only for a fraction of the hundreds of extrinsic corticocortical pathways; the callosal pathway is probably the most studied in this regard (e.g., Houzel et al., 1994; Wang et al., 2008). In contrast, axonal length is readily measured from LM reconstructions typically by the piecewise linear approximation of curvilinear axon trajectories.

In this article, construction cost has referred to the cost of the mature and stable cortical network. So we have not discussed the developmental cost of constructing the mature network or the plasticity cost of remodeling connections of the mature network in response to environmental changes in the adult brain. The task of arranging billions of connections efficiently using developmental mechanisms of axonal and dendritic outgrowth, guidance, branching, and remodeling is immense and appears to require a sophisticated orchestration of molecular cues and gradients as well as activity-dependent modification (Price et al., 2006).

We do not yet know enough about cortical development to determine which cost factors most influence the construction of cortical networks. Developmental chronology may, however, offer some clues. For instance, astroctye and oligodendrocyte cell differentiation lag intrinsic axonal development (Müller, 1992; Bandeira et al., 2009) while the cortical capillary blood supply co-develops with intrinsic axons, most probably guided by common molecular cues (Ben Hamida et al., 1983; Risau, 1997; Tieman et al., 2004). These observations suggest glia and blood vessels may act primarily as supportive rather than strongly constraining factors in the development of cortical networks. The role of oligodendroctyes is of special interest here because their signals are necessary to induce local axon caliber expansion (e.g., Sánchez et al., 1996) yet their number is thought to be regulated by regional axon-derived signals (e.g., Barres and Raff, 1993). Taken together with the developmental chronology, we might infer from this that as axons extend toward their most distant targets it is important to conserve the amount of cellular material used. But later, once axons have reached their targets and are remodeled, thickening selected axonal branches may become more important in order to significantly reduce conduction delays within the network. This example of developmental chronology suggests that the relative influence of cost factors in cortical network formation may vary during development.

Metabolic cost is widely considered as a unifying influence on brain design and function because it is a limited resource essential to an organism's survival (Laughlin and Sejnowski, 2003). A significant proportion of the energy budget is expended on maintaining ionic equilibrium and communicating signals between cortical neurons (e.g., Attwell and Laughlin, 2001). Both construction and routing costs can be defined in energetic terms. The energy required to maintain ionic equilibrium is proportional to the amount of cellular material. The energy required to propagate action potentials and subthreshold signals is related to the path distance a signal must travel along an axon or dendrite from its source to each target.

In summary, we suggest that understanding of the cortical network design problem has been improved by considering construction and routing costs together rather than either by itself. However, a more complete appreciation of cortical network design will require the consideration of other important cost factors such as axon diameter (see Perge et al., 2012).

### Role of cortical dynamics

In this article, we have considered structural communication costs at different scales of cortical organization. But we are keen not to give the impression that we consider dynamics unimportant—clearly signals do not flow unaltered through cortical circuitry and network structure and cortical dynamics are inextricably linked (e.g., Sporns et al., 2000). To generate hypotheses concerning function, structural network analysis examines how information may *potentially* be communicated. Given the considerable complexity of neuronal and synaptic dynamics (Herz et al., 2006), let alone when combined in cortical circuits, structural network analysis offers a simpler alternative for gaining insight into cortical function by generating experimentally testable hypotheses such as the one examined here. It should be remembered that Cajal (1899) made considerable progress in understanding neural communication without being able to record the electrochemical dynamics of the Golgi-stained neurons he studied.

### Scalable brain architecture

Mammalian brains vary greatly in size (see Kaas, 2000). For example, the surface area of primary visual area V1 in humans (2134 mm^2^, Adams et al., 2007) is more than 1500 times larger than that of the mouse (1.40 mm^2^, McCurry et al., 2010). Yet anatomically the substance of cerebral cortex appears similar in many respects. First, neocortical neuronal types such as spiny pyramidal and smooth basket cells are conserved across species (Tyler et al., 1998) though some differences may exist between cortical areas (e.g., Elston, 2003) and between species (e.g., DeFelipe et al., 2003). Second, the ratio of white matter to gray matter volumes of cerebral cortex appears constant across species (Zhang and Sejnowski, 2000). Third, changes in axon diameter and myelination help preserve latency across brain size in corticocortical pathways such as corpus callosum (Wang et al., 2008; Caminiti et al., 2009). Therefore, it is a puzzle how cortical design appears to remain invariant over these dramatic changes of spatial scale (Kaas, 2000; Clark et al., 2001; Stevens, 2001).

The hypothetical principle examined here is consistent with the notion of a scaleable cortical architecture because the trade-off between minimizing spatial and temporal costs should be scale-invariant. In the case of intrinsic axon arbors, for instance, the addition of neuronal branches to increase connectivity would do so without destroying its communication capabilities (see “Axon Arbors”). However, a central issue is whether the balance between communication costs in cortical design is relatively constant, that is, universal or varies according to the particular processing demands of a cortical region or particular niche environment of a species.

### Conclusions

In this article, we reviewed current evidence to evaluate the hypothesis that to optimize communication spatial (construction) and temporal (routing) wiring costs are traded-off across different scales of cortical organization (Budd et al., 2010). We conclude the following:

- At the single neuron scale, a trade-off between spatial and temporal communication costs appears to capture the core morphological structure of axonal and dendritic trees of the most common neuronal types, though this conclusion may not apply for all intrinsic and afferent arbor types. The predictions of the hypothesis can at least be partly validated using existing physiological techniques.
- At the local circuit scale, higher-order statistics obtained from multiple electrode recordings seem to provide a better explanation of network design than those derived from paired recordings. In the absence of complete anatomical reconstructions of local circuits, however, it has not been possible to estimate structural communication costs and test the hypothesis at this scale. Nevertheless, the predictions of the hypothesis might be investigated using fragmentary circuit reconstructions.
- At the pathway scale, corticocortical fiber tracts may also trade-off spatial and temporal communication costs. However, network analysis at this scale is more complicated because there is no standard parcellation scheme and considerable individual variation in corticocortical pathway properties (e.g., fiber density/number and postsynaptic effect on target regions).
- When estimating temporal cost in local circuit and pathway level networks account should be taken of both presynaptic axonal delay and postsynaptic neuronal integration delay, which may be of a similar order of magnitude.
- Recent technical advances in cellular tracing will soon yield massive volumes of data to help evaluate wiring principles of cerebral cortex. To aid hypothesis testing of wiring principles, however, the connectome framework needs to incorporate more morphological data into its description of cortical connectivity.

### Conflict of interest statement

The authors declare that the research was conducted in the absence of any commercial or financial relationships that could be construed as a potential conflict of interest.
